# Impact of the Lectin Chaperone Calnexin on the Stress Response, Virulence and Proteolytic Secretome of the Fungal Pathogen *Aspergillus fumigatus*

**DOI:** 10.1371/journal.pone.0028865

**Published:** 2011-12-07

**Authors:** Margaret V. Powers-Fletcher, Kalyani Jambunathan, Jordan L. Brewer, Karthik Krishnan, Xizhi Feng, Amit K. Galande, David S. Askew

**Affiliations:** 1 Department of Pathology & Laboratory Medicine, University of Cincinnati College of Medicine, Cincinnati, Ohio, United States of America; 2 SRI International, Harrisonburg, Virginia, United States of America; Université de Nice-CNRS, France

## Abstract

Calnexin is a membrane-bound lectin chaperone in the endoplasmic reticulum (ER) that is part of a quality control system that promotes the accurate folding of glycoproteins entering the secretory pathway. We have previously shown that ER homeostasis is important for virulence of the human fungal pathogen *Aspergillus fumigatus*, but the contribution of calnexin has not been explored. Here, we determined the extent to which *A. fumigatus* relies on calnexin for growth under conditions of environmental stress and for virulence. The calnexin gene, *clxA*, was deleted from *A. fumigatus* and complemented by reconstitution with the wild type gene. Loss of *clxA* altered the proteolytic secretome of the fungus, but had no impact on growth rates in either minimal or complex media at 37°C. However, the Δ*clxA* mutant was growth impaired at temperatures above 42°C and was hypersensitive to acute ER stress caused by the reducing agent dithiothreitol. In contrast to wild type *A. fumigatus*, Δ*clxA* hyphae were unable to grow when transferred to starvation medium. In addition, depleting the medium of cations by chelation prevented Δ*clxA* from sustaining polarized hyphal growth, resulting in blunted hyphae with irregular morphology. Despite these abnormal stress responses, the Δ*clxA* mutant remained virulent in two immunologically distinct models of invasive aspergillosis. These findings demonstrate that calnexin functions are needed for growth under conditions of thermal, ER and nutrient stress, but are dispensable for surviving the stresses encountered in the host environment.

## Introduction

*Aspergillus fumigatus* is the causative agent of a life-threatening pulmonary infection that primarily affects the immunocompromised patient population [1]. Current treatment options rely on a small armamentarium of antifungal drugs that are unable to prevent the high mortality rates associated with this infection, particularly in hematopoietic stem cell transplant recipients [2]. Exacerbating this problem are issues of drug toxicity and emerging resistance [3], emphasizing the need for more information on those aspects of fungal physiology that could be interrupted with novel therapies to improve outcome in patients with aspergillosis.

Recent evidence has suggested that fungal pathways that support homeostasis of the endoplasmic reticulum (ER) could represent novel targets for antifungal therapy because of the central role that they play in both virulence and antifungal drug susceptibility. The ER is an interconnected network of endomembranes that promotes the accurate folding of proteins before delivering them to the distal secretory pathway. Maintenance of ER function is accomplished, in part, by a stress signaling pathway known as the unfolded protein response (UPR). The UPR is responsible for activating a program of gene expression to strengthen ER folding capacity when secretion levels are high, or when environmental conditions are not conducive to protein folding [4]. We have previously demonstrated that *A. fumigatus* depends on the master transcriptional regulator of this pathway, HacA, for the expression of full virulence [5], [6]. This suggests that the fungus is under ER stress in the mammalian host and needs the UPR to sustain the infection by restoring homeostatic balance to the secretory pathway. Similar findings were made in *Alternaria brassicicola*, a necrotrophic plant pathogen that kills host cells through the secretion of numerous enzymes and toxins. Deletion of *A. brassicicola* HacA decreased the secretory capacity of the fungus, resulting in impaired virulence and increased susceptibility to plant antimicrobial metabolites [7]. Notably, the rice blast fungus *Magnaporthe oryzae* has been shown to rely on the ER chaperone LHS1 for its virulence [8]. Because LHS1 is only one component of the entire UPR stress response [9], [10], this suggests that individual chaperones could mediate the effects of the UPR on virulence.

Calnexin is an ER membrane-bound lectin chaperone that is one of the major targets of the UPR during ER stress [11], [12], [13]. The protein is part of an ER quality control system known as the calnexin cycle [14]. In metazoans, two key chaperones participate in the calnexin cycle; calnexin itself, a type 1 transmembrane protein, together with calreticulin, a soluble homolog of calnexin [15]. However, only calnexin has been identified in fungal species [16], [17]. Functional studies have revealed that calnexin promotes folding by binding to the N-linked glycans that are added to nascent polypeptides as they enter the ER, thereby preventing aggregation. This glycoprotein-calnexin interaction undergoes cycles of release and re-binding until the glycoprotein achieves its native conformation, after which the protein is released for secretion into the distal secretory pathway [14]. In this study, we examined the contribution of calnexin to stress responses that would be encountered by *A. fumigatus* in its native environment as well as the host. Although *clxA* was dispensable for most aspects of *A. fumigatus* physiology, it was required under conditions of thermal, ER and nutrient stress. The virulence of the Δ*clxA* mutant was indistinguishable from that of wild type (wt) *A. fumigatus* however, indicating that *clxA*-dependent functions are largely dispensable for infection of the host.

## Results and Discussion

### Construction of a calnexin-deficient strain of *A. fumigatus*

**Figure 1:**
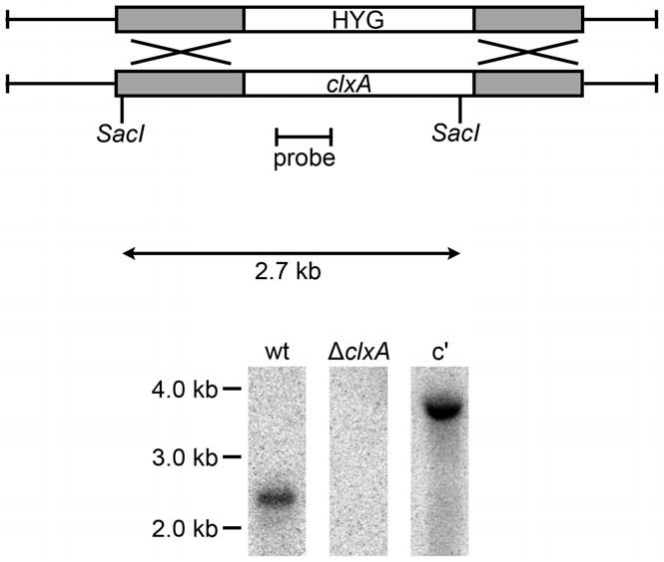
Deletion of calnexin from *A. fumigatus*. The *clxA* gene was deleted by replacing the coding region with the hygromycin resistance cassette (HYG). Southern blot analysis of *SacI*-digested genomic DNA using a probe located with the *clxA* coding region identified the predicted 2.7 kb fragment in wt *A. fumigatus*, which was not present in the Δ*clxA* mutant. The portion of the calnexin gene that was used to generate the complemented strain (C′) contains a single internal *SacI* site, so the 3.7 kb band evident with this probe reflects a single ectopic integration of the reintroduced *clxA* gene.

The *A. fumigatus* calnexin mRNA (Genbank accession: AY560606) encodes a protein with the same two sets of repeated peptide motifs that are characteristic of the calreticulin/calnexin family, together with a predicted membrane-spanning domain (Figure S1). The protein is most closely related to that of other filamentous fungi, but among yeast species it is more closely related to the *Schizosaccharomyces pombe* ortholog (46% identity) than to the *Saccharomyces cerevisiae* ortholog (28% identity). A calnexin deficient strain of *A. fumigatus* was constructed by replacing the gene (*clxA*) with a hygromycin resistance cassette (Figure 1 and S2). The Δ*clxA* mutant was viable and grew normally on either minimal or rich medium (data not shown). This contrasts the essentiality of calnexin in *S. pombe* [18], but is similar to the viability of calnexin mutants reported in *A. niger* [12], *Aspergillus oryzae* [19] and *S. cerevisiae* [20].

### Loss of calnexin alters the proteolytic secretome of *A. fumigatus*

**Figure 2:**
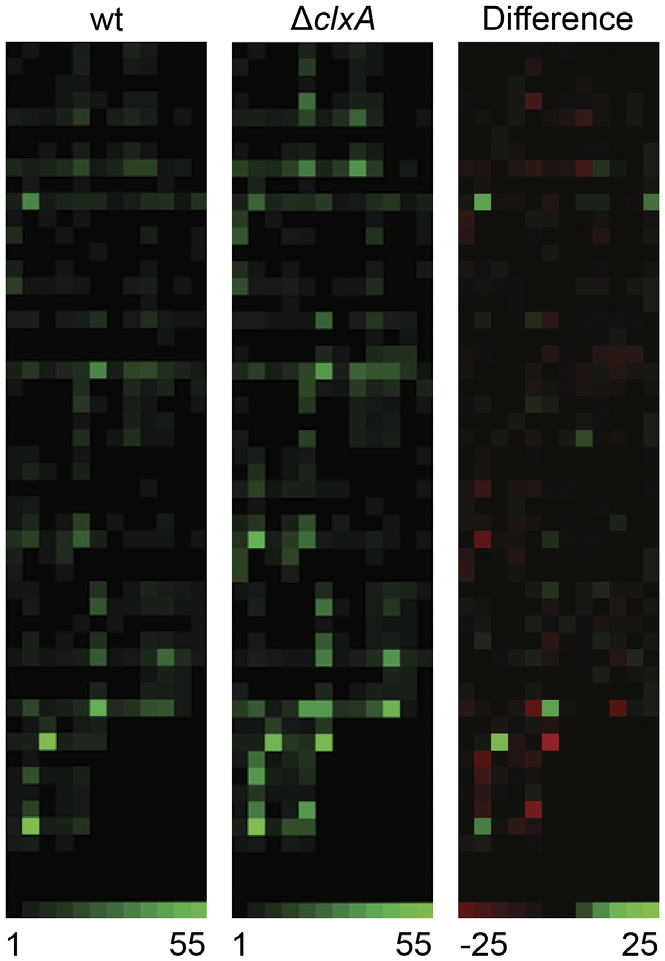
Loss of calnexin alters proteolytic secretion. Secreted protease activity in culture supernatants was profiled using a library of fluorescence resonance energy transfer (FRET) labeled peptide substrates, as described in Materials and Methods. Equimolar mixtures of up to 8 individual FRET peptides in each well of duplicate microtiter plates were incubated with culture supernatants from the indicated strains and heat maps were generated from the average fluorescent signals generated by substrate cleavage, with each square corresponding to a single assay well. Panel 1: wt *A. fumigatus*. Panel 2: *ΔclxA* mutant. Panel 3: Relative change in substrate specificity profile expressed as the difference of normalized fold change values in panels 1 and 2 (wt minus *ΔclxA*). Wells containing substrates with greater cleavage in Δ*clxA* supernatants relative to wt supernatants are shown in red. The range of fold change values used to generate the wt and Δ*clxA* heat maps is 1–55 and the range of values used to generate the subtracted heat map (difference) is −25 to +25.

Previous studies in yeast have shown that loss of calnexin function is associated with increased secretion of some proteins [21], [22], [23], [24]. In this study, we used a more comprehensive approach to determine how loss of calnexin would affect the proteolytic secretome of *A. fumigatus*, using a combinatorial library of internally quenched fluorogenic peptide substrates [25]. This library is comprised of a panel of up to eight individual fluorogenic peptides in each well of a microtiter plate. When the substrates are cleaved, a fluorophore is liberated from a quenching moiety, resulting in a fluorescence signal that is proportional to the extent of cleavage. *A. fumigatus* culture supernatants were used to screen the library as described in Materials and Methods, and heat maps were generated from the resulting data. As shown in Figure 2, loss of *clxA* altered the secreted proteolytic signature of the fungus, with a remarkably high number of substrates showing increased cleavage by Δ*clxA* supernatants relative to wt (indicated by the red squares). However, this was not associated with any changes in the ability of the mutant to grow on a complex protein source such as skim milk or fetal bovine serum (data not shown). The precise mechanism by which loss of calnexin increases the secretion of some proteins in both yeast and *A. fumigatus* is not yet clear. However, since part of calnexin's role in protein quality control is to retain incompletely folded proteins in the ER until they achieve the appropriate conformation [14], the loss of this retention function may allow for more rapid secretion of proteins that would ordinarily take longer to traffic through the secretory pathway. It is possible that some of these prematurely released proteins could be partially unfolded. However, given the large number of functional proteases identified in Δ*clxA* supernatants in this study, it appears that redundant mechanisms of protein folding in the ER can adequately compensate for loss of calnexin and ensure that secreted proteases achieve the appropriate conformation for functionality.

### Calnexin facilitates growth under conditions of thermal stress

**Figure 3:**
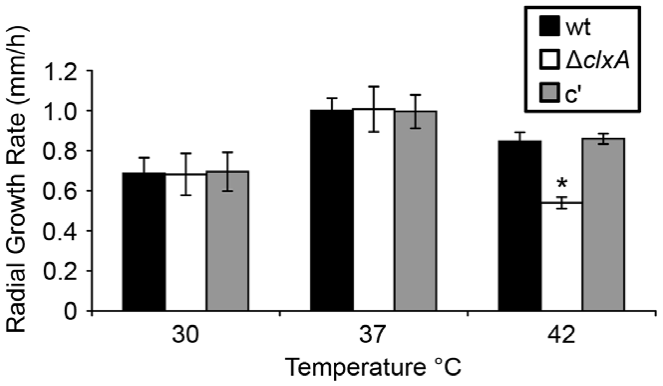
Calnexin is required for thermotolerant growth. Equal numbers of conidia were plated onto the center of an IMA plate and incubated for 3 days at the indicated temperatures. Values represent the average radial growth rate (mm/h) from three individual experiments ± SD. *Statistically significant by Student's T-test (p<0.001).

In nature, *A. fumigatus* resides in composting material, an environment that undergoes wide fluctuations in temperature because of intense microbial activity [26]. *A. fumigatus* has responded to the thermal selection pressure in this environmental niche by evolving mechanisms of thermotolerance that allow the fungus to thrive at temperatures up 60° C, with an optimum between 37°C and 42°C [27], [28]. As shown in Figure 3, the Δ*clxA* mutant grew normally at temperatures up to 37°C, but showed a 45% reduction in growth rate at 42°C indicating a role for calnexin in the thermotolerance of this fungus. We have previously shown that loss of UPR signaling by deletion of *hacA* creates a cell wall defect that reduces thermotolerance by increasing hyphal fragility at higher temperatures, resulting in tip lysis [6]. The Δ*clxA* mutant showed no evidence of hyphal tip fragility at temperatures up to 50°C however (data not shown), indicating that *A. fumigatus* can maintain cell wall integrity at high temperatures in the absence of calnexin. Moreover, although calnexin has been previously implicated in cell wall synthesis in *S. cerevisiae* [29], the *A. fumigatus* Δ*clxA* mutant showed wt sensitivity to multiple cell wall stressors, including calcofluor white, nikkomycin, Congo red or caspofungin (Figure S3 and S4). While these data do not eliminate the possibility that calnexin contributes to cell wall homeostasis in *A. fumigatus*, they suggest that any change in cell wall composition caused by loss of calnexin is relatively minor and unlikely to account for the heightened thermosensitivity of the Δ*clxA* mutant. Since protein folding is temperature dependent [30], we speculate that the chaperone function of calnexin promotes the folding of one or more client proteins that are needed for optimal growth at elevated temperatures. Alternatively, calnexin could increase overall fitness by preventing the toxic accumulation of misfolded proteins in the ER that may arise as a result of thermal stress.

### Calnexin facilitates growth under conditions of acute protein folding stress

**Figure 4:**
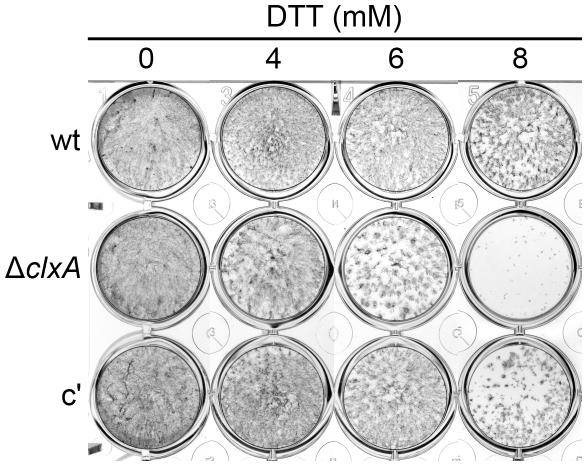
Calnexin promotes growth under conditions of acute ER stress. Equal numbers of conidia were added to individual wells of a 24-well plate containing liquid AMM and the indicated concentrations of DTT. Plates were incubated at 37°C for 3 days, after which the mycelial biomass that was adhered to the plate surface was stained with methylene blue and photographed.

Calnexin mRNA levels are increased by the UPR under conditions of acute ER stress, suggesting a role for this chaperone in the response to unfolded proteins [11], [12], [13], [31]. To test this, we compared growth in the presence of dithiothreitol (DTT), a reducing agent that induces the UPR by disrupting the disulfide bonds that are necessary for protein folding [32]. As shown in Figure 4, the Δ*clxA* mutant was unable to grow in concentrations of DTT that had minimal effects on the growth of the wt and complemented strains. This is similar to what has been described in a calnexin mutant of *A. oryzae* [19], and supports a role for calnexin under conditions that induce acute ER stress. However, this finding contrasted the effects of the ER stress-inducing agents tunicamycin (TM) and brefeldin A (BFA), neither of which showed differential activity against the *A. fumigatus* Δ*clxA* mutant (data not shown). The inability of calnexin deletion to alter sensitivity to TM or BFA is likely due to the different mechanisms by which these agents induce ER stress. For example, TM increases the level of misfolded proteins in the ER by interfering with the N-linked glycosylation that is necessary for accurate protein folding [32]. Since calnexin binds N-linked glycans on nascent polypeptides as they begin to fold in the ER, TM may mask the effects of calnexin by interfering with the assembly of the glycan precursor on these proteins. Similarly, BFA induces ER stress by interfering with ER-Golgi transport [33], which is a relatively late step in the secretory pathway where calnexin function may have less influence. The ability of the *ΔclxA* mutant to grow normally in the presence of TM and BFA contrasts the *ΔhacA* mutant, which is highly sensitive to these compounds [6]. This reflects the dominant role that HacA plays as the master regulator of the UPR, as opposed to the more specialized role of calnexin as one of several ER chaperones that are downstream of UPR signaling.

### Calnexin facilitates growth under starvation conditions

**Figure 5:**
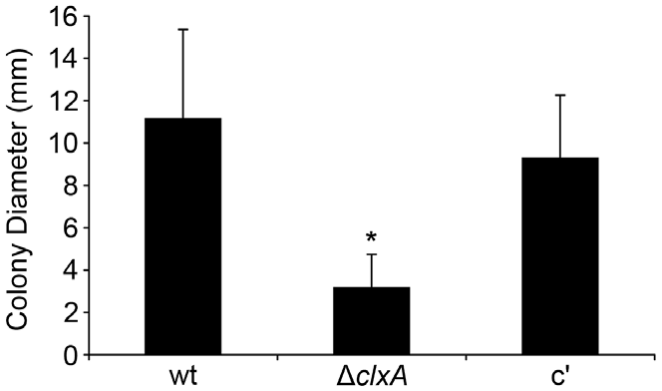
Calnexin is required under nutrient starvation conditions. Agar plugs containing hyphae from overnight cultures of conidia plated onto YG plates were transferred to starvation medium (1% agarose in sterile distilled water) and colony diameters were measured after 7 days at 37°C. Values represent the average of 9 biological replicates ± SD. *Statistically significant by Student's T-test (p<0.001).

An increasing body of evidence suggests that *A. fumigatus* is under nutrient stress in the host environment and must undergo metabolic changes to adapt to these conditions [34]. To determine whether calnexin contributes to this process, the Δ*clxA* mutant was tested for its ability to grow under nutrient limiting conditions. Unlike wt *A. fumigatus* hyphae, which can support a limited amount of growth in the absence of extracellular nutrients [35], the Δ*clxA* mutant was unable to grow under these conditions, suggesting a role for calnexin in the adaptive response to acute starvation stress (Figure 5). This phenotype is reminiscent of the *A. fumigatus* Δ*atg1* mutant, which is deficient in a serine kinase required for autophagy [35]. Autophagy is a catabolic pathway that employs a complex membrane trafficking system to degrade intracellular constituents into usable nutrients during periods of nutrient deprivation [36]. Although the exact role that calnexin plays in the response to starvation is presently unknown, it is intriguing to speculate that autophagy components are important clients of the calnexin cycle in *A. fumigatus*.

### Calnexin is required for growth in cation-depleted medium

**Figure 6:**
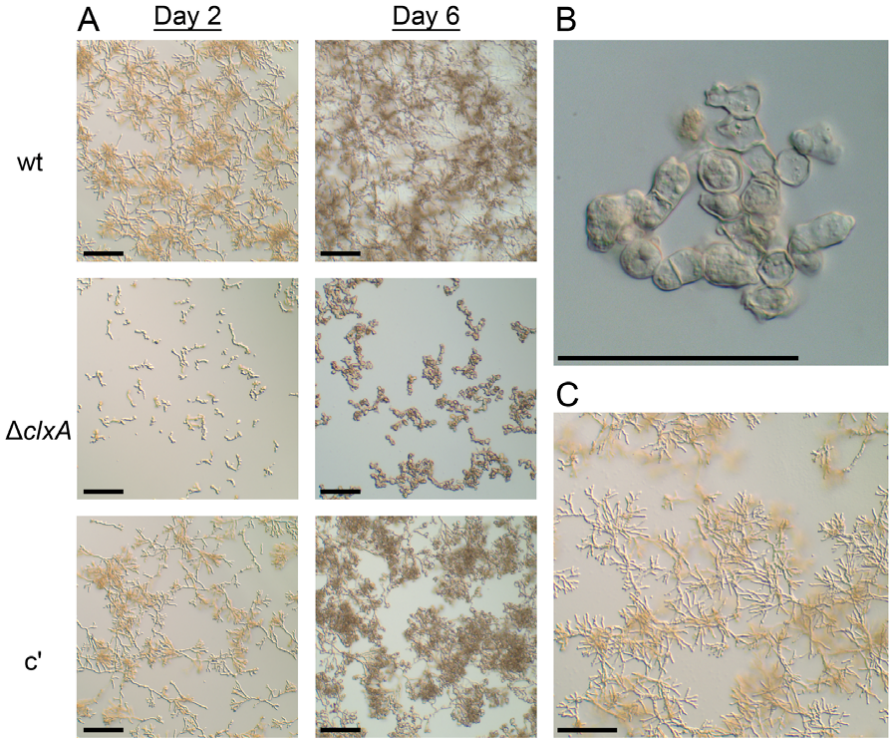
Calnexin promotes growth in cation-depleted medium. Equal numbers of conidia from the indicated strains were inoculated onto glass coverslips in liquid AMM containing 1 mM EDTA (AMM-EDTA) and incubated at 37°C. (A) Impaired germination of Δ*clxA* conidia in EDTA: coverslips were removed after 2 and 6 days incubation and fungal morphology was photographed by differential interference contrast microscopy. (B) Abnormal morphology of the Δ*clxA* mutant after prolonged incubation in EDTA: A high power image of the abnormally swollen conidia and hyphae of the Δ*clxA* mutant after 6 days of incubation in AMM-EDTA at 37°C is shown. (C) Supplementation with zinc rescues the growth of Δ*clxA* in AMM-EDTA: The Δ*clxA* conidia were inoculated into AMM-EDTA medium supplemented with 500 µM ZnS0_4_ and cultured for 2 days at 37°C.

Sequestration of iron or zinc is a major mechanism through which the host inhibits microbial growth, and the ability of *A. fumigatus* to adapt to iron or zinc limitation is an established virulence mechanism for this fungus [37], [38]. To determine whether calnexin impacts this adaptive response, we compared the ability of conidia to germinate in medium that was depleted of metal ions by the addition of the chelating agent ethylenediaminetetraacetic acid (EDTA). The germination of *A. fumigatus* conidia begins with a period of isotropic expansion (swelling), followed by the elaboration of a germ tube and the establishment of polarized hyphal growth. The germination rates of wt and Δ*clxA* conidia were indistinguishable in the absence of EDTA (data not shown). In the presence of EDTA, wt conidia were able to germinate into hyphae within two days and had already started to branch (Figure 6A). This contrasted the Δ*clxA* mutant, which had only formed small germlings at the same time point, indicating a delay in germination (Figure 6A, day 2). Unlike wt, the Δ*clxA* germlings were unable to sustain polarized growth upon further incubation, resulting in abnormally swollen hyphae with irregular morphology (Figure 6B). Supplementation with an excess of Zn^2+^ or Fe^2+^ fully rescued the ability of Δ*clxA* conidia to elaborate hyphae at this concentration of EDTA (Figure 6C and data not shown), consistent with metal ion deficiency as the cause of this phenotype. The increased sensitivity of Δ*clxA* to EDTA may reflect the existence of specific calnexin client proteins involved in metal ion homeostasis, such as membrane transporters or zinc-finger transcription factors. Alternatively, since many ER functions are metal ion-dependent, it may be more difficult for a metal ion-depleted ER to tolerate the loss of calnexin functions. The observation that EDTA induces the UPR is consistent with this latter possibility [39].

### Calnexin is dispensable for *A. fumigatus* virulence

**Figure 7:**
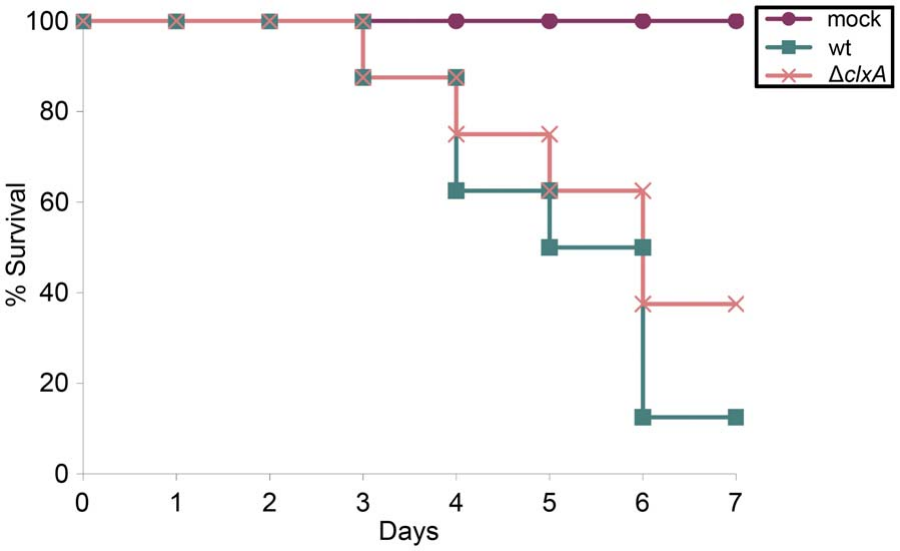
Calnexin is dispensable for *A. fumigatus* virulence. Groups of 8 CF-1 outbred mice were immunosuppressed with a single dose of triamcinolone acetonide on day −1 and infected intranasally with conidia from the indicated strains on day 0. Mortality was monitored for 7 days. The virulence of the Δ*clxA* mutant was statistically indistinguishable from that of wt.

The virulence of the Δ*clxA* mutant was tested in two distinct mouse models of invasive aspergillosis that differ in the extent of immunosuppression; a corticosteroid model that involves transient immunosuppression with a single-dose of triamcinolone acetonide and a neutropenic model that involves a prolonged immunosuppression regimen that pairs neutrophil depletion with corticosteroid-induced immunosuppression. As shown in Figure 7, the loss of *clxA* has little-to-no effect on the virulence of *A. fumigatus* in the corticosteroid model. Similar results were obtained using the neutropenic model (Figure S5). Histopathologic analysis of lung tissue in the neutropenic model confirmed that fungal growth and inflammation were comparable in both wt- and Δ*clxA*-infected mice (Figure S6). Combined, these results indicate that calnexin functions are dispensable for surviving the major environmental stresses that are encountered in the mammalian host. This finding contrasts the situation in the plant fungal pathogen *M. oryzae*, where calnexin is required for the elaboration of a specialized infection structure called an appressorium that is essential for virulence [40]. *A. fumigatus* does not form these structures however, which may account for the different requirements for calnexin in the pathogenicity of these diverse fungal pathogens.

### Summary

*A. fumigatus* is normally found in compost, a harsh environment that challenges the fungus to tolerate wide fluctuations in temperature, nutrient availability and the toxic effects of compounds released by competing microbes. Our data reveal that calnexin protects *A. fumigatus* from the adverse effects of high temperature, nutrient deprivation and toxins that disrupt ER homeostasis. This suggests that calnexin has important functions that contribute to the ability of *A. fumigatus* to thrive in its ecological niche of decaying organic debris. However, our data demonstrate that calnexin is dispensable for infection of a mammalian host, suggesting that redundant pathways of ER homeostasis are sufficient to support the virulence of this organism. Further work is needed to identify these pathways and determine how they cooperate with calnexin to meet the challenge of protein folding in a fungus that is highly adapted for secretion.

## Materials and Methods

### Strains and culture conditions

Strains were maintained on *Aspergillus* Minimal Medium (AMM) [41] containing 0.01 M ammonium tartrate as the nitrogen source. The wild type strain used was H237, a clinical isolate. Unless otherwise noted, all experiments were conducted at 37°C to recapitulate in vivo growth conditions. Thermotolerance was assessed by inoculating 5,000 conidia into the center of a plate of rich medium (Inhibitory Mold Agar, IMA) and radial growth was monitored for 3 days at different temperatures. For analysis of DTT susceptibility, 5,000 conidia were inoculated into each well of a 24-well plate containing liquid AMM supplemented with different concentrations of DTT. Plates were incubated at 37°C for 3 days without shaking. The medium was aspirated, and the hyphae adhering to the base of the well were stained with 0.5% (w/v) methylene blue for 1 hour at 37°C. After removing the methylene blue solution the adherent hyphae were rinsed with sterile water and dried prior to photographing. Sensitivity to tunicamycin (10–100 µg/ml) and brefeldin A (5–15 µg/ml) was determined by spotting conidia onto the center of a plate of AMM containing the drug and monitoring radial growth for 2–4 days at 37°C. Caspofungin susceptibility was determined using the Etest antifungal susceptibility kit (AB BIODISK) according to the manufacturer's instructions, with the following modifications. One million conidia were spread evenly onto the surface of a 150 mm plate of IMA agar using a glass rod. The inoculated agar surface was allowed to dry for approximately one hour before Etest strips containing caspofungin were applied. The plates were incubated at 37°C for 24 hours. The minimal inhibitory concentration was read as the lowest drug concentrations at which the border of the elliptical inhibition zone intercepted the scale on the antifungal strip. Sensitivity to Congo red (CR, 25–150 µg/ml), nikkomycin (50–250 µg/ml), or calcofluor white (CFW, 5–35 µg/ml) was determined by spotting 2,000 conidia onto the center of a plate of IMA containing the compound and monitoring radial growth for 24 hours (CR and CFW) or 36 hours (Nikkomycin) at 37°C.

Growth under starvation conditions was determined as previously described [35]. Briefly, 200 conidia were spread onto the surface of a YG plate (0.5% yeast extract and 2% glucose) and incubated overnight at 37°C. Hyphal plugs containing individual colonies were obtained using the tip of a sterile glass pasture pipette and transferred onto the center of a plate of water/agarose medium (1% agarose in sterile deionized distilled water) and the extent of radial growth was monitored after 7 days of incubation at 37°C.

For analysis of growth under conditions of metal ion depletion, 3×10^4^ conidia were inoculated onto sterile glass coverslips submerged in 3 mL of AMM containing 1 mM EDTA and incubated at 37°. Coverslips were removed after 2 and 6 days incubation, rinsed with sterile water, and photographed by differential interference contrast microscopy.

### Deletion and reconstitution of the *A. fumigatus clxA* gene

PCR primers used in the study are listed in Table 1. Total RNA was extracted from overnight cultures of wt *A. fumigatus* by crushing the mycelium in liquid nitrogen and resuspending in TRI reagent LS (Molecular Research Center, Cincinnati, OH). The RNA was then reverse-transcribed using the Superscript II reverse transcriptase first-strand synthesis system (Invitrogen) and PCR amplified using primers 639 and 640.

**Table 1:** PCR primers used in this study.

Primer	Sequence (5′-3′)
395	CTCCATACAAGCCAACCACGG
396	CGTTGCAAGACCTGCCTGAA
398	CGCCAGGGTTTTCCCAGTCACGACAAGTGGAAAGGCTGGTGTGC
399	AGCGGATAACAATTTCACACAGGATCGCGTGGAGCCAAGAGCGG
632	GATGCTTCTTGTCAGTATCCT
633	GTCGTGACTGGGAAAACCCTGGCGGCTAATACCCGAGATCTCTG
634	TCCTGTGTGAAATTCTTATCCGCTGGATACGGGCGAATAATACG
635	CAAAGGACGACGATGTTGTT
636	TACGTCGGTGAATGGGCTGT
637	AGGCGCTGTCATGTGCTTCT
639	CAGAGATCTCGGGTATTAGC
640	CGTATTATTCGCCCGTATC
641	GGCTTTCGACAGAACATTGG
672	AGGTCCCGTCATCTATTTCC

M13-derived sequences used for overlap PCR are underlined.

The calnexin gene was replaced with the hygromycin resistance gene using the split-marker method [42]. The first two-thirds of the hygromycin resistance cassette were amplified from pAN7-1 using primers 398 and 395, creating PCR product #1. The second two-thirds of hygromycin were then amplified with primers 396 and 399, creating PCR Product #2. The left arm of the *clxA* gene was amplified from wt DNA using primers 632 and 633, and the right arm was amplified with primers 634 and 635, generating PCR products #3 and #4, respectively. PCR products #1 and #3 were then combined in an overlap PCR reaction with primers 632 and 395 to generate PCR product #5 and PCR products #2 and #4 were combined in an overlap reaction with primers 396 and 635 to generate PCR product #6. PCR products #5 and #6 were then cloned into pCR-Blunt II-TOPO (Invitrogen) to create plasmids p569 and p568, respectively. The p569 and p568 plasmids were linearized with *NsiI* and *EcoRI*, respectively, and transformed into *A. fumigatus* protoplasts as previously described [43]. Loss of the *clxA* gene was confirmed by Southern blot analysis of genomic DNA isolated from hygromycin resistant monoconidial isolates using an internal probe that was PCR-amplified from wt genomic DNA using primers 636 and 637 (Figure 1), as well as an upstream probe that was PCR-amplified from wt genomic DNA using primers 632 and 633, corresponding to the left arm of the calnexin-deletion cassette (Figure S2).

To construct the *clxA* complementation plasmid, the *clxA* gene including 472 bp upstream of the ATG was PCR-amplified from wt genomic DNA using primers 641 and 672 and cloned into pCR-Blunt II-TOPO (Invitrogen) to create p575. A phleomycin resistance cassette was then excised from plasmid 565 and inserted into p575 to create p612. Plasmid 612 was linearized with *XbaI*, transformed into Δ*clxA* protoplasts, and stable integrants were selected on plates containing phleomycin. Ectopic reconstitution of the *clxA* gene was confirmed by genomic Southern blot analysis of phleomycin-resistant monoconidial isolates using the internal *clxA* probe shown in Figure 1 and the upstream probe shown in Figure S2.

### Analysis of protease secretion by substrate specificity profiling

Conidia were inoculated to a concentration of 1×10^5^ conidia/ml in 60 mL of AMM supplemented with 10% heat-inactivated human AB serum (Innovative Research). After incubating at 37°C for 72 h at 150 rpm, the mycelium was removed by filtration and each culture supernatant was diluted 1∶50 in sterile-filtered HEPES buffer (50 mM HEPES, 100 mM NaCl, 10 mM CaCl_2_, pH 8.0). These dilutions were found to give comparable fluorescence intensity values in preliminary experiments. A FRET peptide library comprised of 512 microtiter plate wells, each containing an equimolar mixture of up to 8 individual peptides, was obtained (Mimotopes, Clayton, Australia) [25]. Each well (50 nmol of peptide) was dissolved in 100 µL of 50% acetonitrile in ultrapure water. This solution (5 µL/well) was transferred to low volume black microtiter plates (Molecular Devices, Sunnyvale, CA) containing 20 µL of HEPES buffer. Diluted fungal culture supernatant (20 µL per well) was added to each well. Time-resolved fluorescence data were obtained on an Analyst HT instrument (Molecular Devices) using excitation and emission filters of 320 nm and 420 nm, respectively. The fluorescence intensity fold change after 5 hr at room temperature was calculated as F_final_/F_initial_ and each data set was normalized to the highest global signal intensity. No fluorescence was observed in control culture medium lacking fungal supernatant. Heat maps were generated from these data in which each square corresponds to a single assay well (Heatmap Builder, Ashley Lab, Stanford) [44].

### Animal models of invasive aspergillosis

For the corticosteroid immunosuppression model, groups of 8 CF-1 outbred female mice were given a single dose of the synthetic corticosteroid triamcinolone acetonide (40 mg/kg of body weight injected subcutaneously) on day −1. On day 0, the mice were anaesthetized with 3.5% isofluorane and inoculated intranasally with a 20 µL saline suspension containing 2×10^5^ conidia from wt or the Δ*clxA* mutant, or with 20 µl of a 0.9% sodium chloride solution for a mock infection control (4 mice). Mortality was monitored for 7 days, and statistical significance was assessed by the log-rank test using Sigma Stat 3.5.

For the neutropenic model, groups of 12 CF-1 outbred female mice were immunosuppressed by intraperitoneal injection of cyclophosphamide (150 mg/kg) on days −2 and +3, as well as subcutaneous injections of triamcinolone acetonide (40 mg/kg) on days −1 and +6. Mice were inoculated with 2×10^5^ conidia and mortality was monitored for 14 days. Statistical significance was assessed using the Sigma Stat 3.5 log-rank test.

For histopathologic analysis, CF-1 outbred female mice were immunosuppressed according to the neutropenic model described above, infected with 2×10^5^ conidia, and sacrificed on day +3. The lungs were fixed by inflation with 4% phosphate-buffered paraformaldehyde, dehydrated and embedded in paraffin, sectioned at 5 µm, and stained with hematoxylin and eosin (HE) or Grocott methenamine silver (GMS). Microscopic examinations were performed on an Olympus BH-2 microscope and imaging system using Spot software version 4.6.

### Animal ethics statement

Animal experiments were carried out in strict accordance with the Guide for the Care and Use of Laboratory Animals, the Public Health Service Policy on the Humane Care and Use of Laboratory Animals and all U.S. Animal Welfare Act Regulations. The experiments were approved by the Institutional Animal Care and Use Committee of the University of Cincinnati (protocol # 06-01-03-02). All efforts were made to minimize animal suffering.

### Genbank accession numbers

*A. fumigatus clxA* gene (XM_746454.1), *A. fumigatus clxA* mRNA (AY560606.1).

## Supporting Information

Figure S1 **Multiple sequence alignment of calnexin orthologs.** The *A. fumigatus* calnexin protein (Af; XP_751547) is compared to orthologs from *A. niger* (An; AJ299945), *S. pombe* (Sp; P26581), *H. sapiens* (Hs; P27824), and *S. cerevisiae* (Sc; P27825). Black boxes denote identical amino acids, whereas grey boxes denote similar amino acids. The sequence was aligned using DNAMAN software (Lynnon Corp, Canada) using default parameters. Results were exported in CLUSTALW format for shading using BOXSHADE 3.21 (http://www.ch.embnet.org/software/BOX_form.html). The two sets of repeated peptide motifs (1–4) that are characteristic of the calreticulin/calnexin family are shown by the brackets. The asterisk denotes the transmembrane domain of *A. fumigatus* calnexin predicted by TMHMM Server v. 2.0 (http://www.cbs.dtu.dk/services/TMHMM-2.0).
(TIF)

Figure S2 **Deletion of calnexin from** ***A. fumigatus*** **.** Southern blot analysis of *BamH*I-digested genomic DNA using a flanking probe located upstream of the *clxA* gene was used to confirm calnexin gene deletion. Replacement of the *clxA* gene with the hygromycin resistance cassette introduced a *BamH*I site that reduced a 5.6 kb wt fragment to the expected 3.7 kb. Two closely migrating bands above 5.6 kb were also evident in the Δ*clxA* mutant, indicating the presence of at least two ectopic integrations of the disruption cassette. The complemented strain (C′) contains a single ectopic integration of the *clxA* gene, which is evident by the unique 5.0 kb band that is smaller than the wt 5.6 kb band because it lacks the flanking *BamHI* sites.
(TIF)

Figure S3 **Loss of calnexin does not increase sensitivity to caspofungin.** Caspofungin sensitivity was determined using the Etest method. Etest strips containing caspofungin were applied to IMA plates inoculated with equal amounts of conidia. The plates were incubated at 37°C for 24 hours. The lowest drug concentrations at which the border of the elliptical zone of inhibition intercepted the scale on the antifungal strip (MIC) was indistinguishable between the strains, indicating that loss of calnexin did not alter caspofungin sensitivity. In addition, fungal growth was evident within the zone of inhibition in all three strains, consistent with the known fungistatic effects of this drug against *A. fumigatus*.
(TIF)

Figure S4 **Calnexin is not required under cell wall stress conditions.** Sensitivity to Congo red (CR), nikkomycin, or calcofluor white (CFW) was determined by spotting equal amounts of conidia onto the center of a plate of IMA containing each compound at the indicated concentrations and monitoring radial growth for 24 hours (CR and CFW) or 36 hours (Nikkomycin) at 37°C. The Δ*clxA* strain phenocopies wt at all concentrations of each cell wall stress-inducing agent.
(TIF)

Figure S5 **Calnexin is dispensable for** ***A. fumigatus*** **virulence.** Groups of 12 CF-1 outbred mice were immunosuppressed with cyclophosphamide and triamcinolone acetonide and inoculated with 2×10^5^ conidia as described in Materials and Methods. Pulmonary fungal infections were confirmed in all mice that died by plating lung tissue for fungal growth. One of the four mock-infected control mice died on day +10 of a bacterial infection. The virulence of the Δ*clxA* mutant was statistically indistinguishable from that of wt.
(TIF)

Figure S6 **Histopathology of infected lung tissue.** Using the neutropenic immunosuppression model, mice were infected with 2×10^5^ conidia in a separate experiment and sacrificed on day +3, as described in Materials and Methods. The lungs were sectioned at 5 µm and stained with hematoxylin and eosin (HE) or Grocott methenamine silver (GMS). Comparable levels of fungal growth and inflammation were observed in both wt- and Δ*clxA*-infected mice, resulting in similar amounts of bronchiolar erosion and migration of the hyphae across the airway wall. Microscopic examinations were performed on an Olympus BH-2 microscope and imaging system using Spot software version 4.6. Scale bar represents 100 µm.
(TIF)
