# Supplementary figures and images for: Impact of the Lectin Chaperone Calnexin on the Stress Response, Virulence and Proteolytic Secretome of the Fungal Pathogen Aspergillus fumigatus
Source: PLoS One. 2011 Dec 7;6(12):e28865. doi: 10.1371/journal.pone.0028865 (PMC3233604; doi:10.1371/journal.pone.0028865)

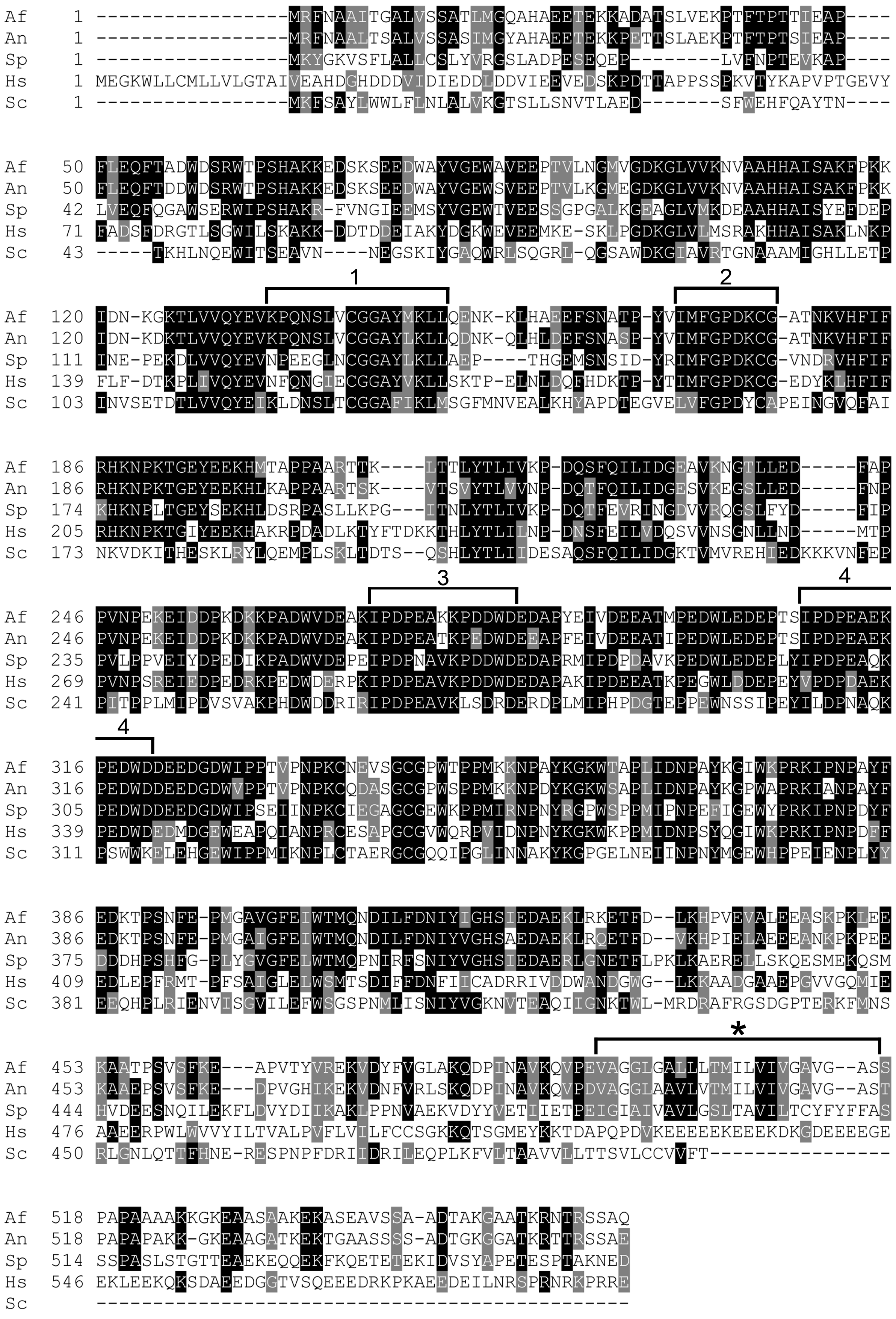

Supplement: Figure S1 — Multiple sequence alignment of calnexin orthologs. The A. fumigatus calnexin protein (Af; XP_751547) is compared to orthologs from A. niger (An; AJ299945), S. pombe (Sp; P26581), H. sapiens (Hs; P27824), and S. cerevisiae (Sc; P27825). Black boxes denote identical amino acids, whereas grey boxes denote similar amino acids. The sequence was aligned using DNAMAN software (Lynnon Corp, Canada) using default parameters. Results were exported in CLUSTALW format for shading using BOXSHADE 3.21 (http://www.ch.embnet.org/software/BOX_form.html). The two sets of repeated peptide motifs (1–4) that are characteristic of the calreticulin/calnexin family are shown by the brackets. The asterisk denotes the transmembrane domain of A. fumigatus calnexin predicted by TMHMM Server v. 2.0 (http://www.cbs.dtu.dk/services/TMHMM-2.0). (TIF) [file pone.0028865.s001.tif]

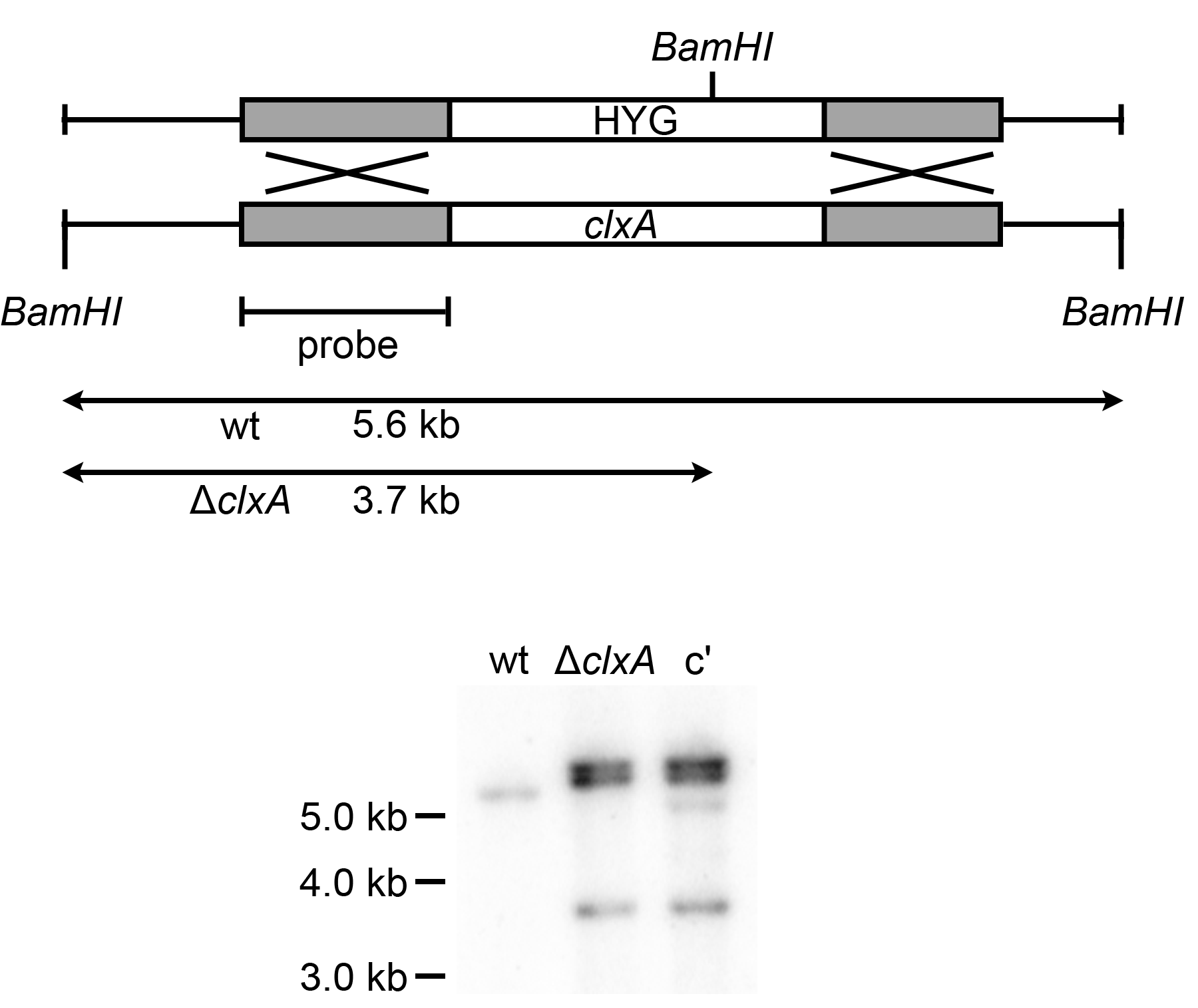

Supplement: Figure S2 — Deletion of calnexin from A. fumigatus . Southern blot analysis of BamHI-digested genomic DNA using a flanking probe located upstream of the clxA gene was used to confirm calnexin gene deletion. Replacement of the clxA gene with the hygromycin resistance cassette introduced a BamHI site that reduced a 5.6 kb wt fragment to the expected 3.7 kb. Two closely migrating bands above 5.6 kb were also evident in the ΔclxA mutant, indicating the presence of at least two ectopic integrations of the disruption cassette. The complemented strain (C′) contains a single ectopic integration of the clxA gene, which is evident by the unique 5.0 kb band that is smaller than the wt 5.6 kb band because it lacks the flanking BamHI sites. (TIF) [file pone.0028865.s002.tif]

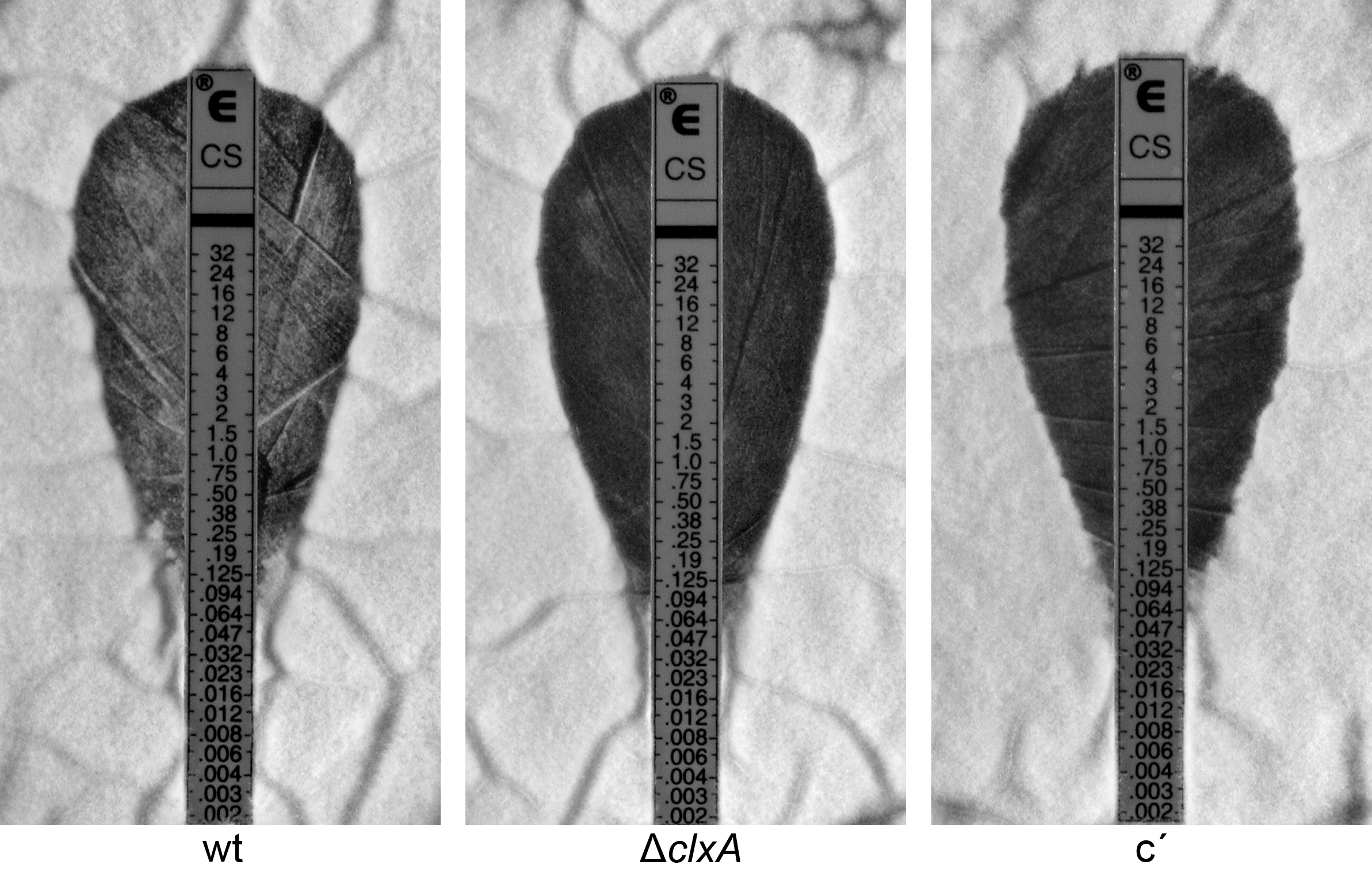

Supplement: Figure S3 — Loss of calnexin does not increase sensitivity to caspofungin. Caspofungin sensitivity was determined using the Etest method. Etest strips containing caspofungin were applied to IMA plates inoculated with equal amounts of conidia. The plates were incubated at 37°C for 24 hours. The lowest drug concentrations at which the border of the elliptical zone of inhibition intercepted the scale on the antifungal strip (MIC) was indistinguishable between the strains, indicating that loss of calnexin did not alter caspofungin sensitivity. In addition, fungal growth was evident within the zone of inhibition in all three strains, consistent with the known fungistatic effects of this drug against A. fumigatus. (TIF) [file pone.0028865.s003.tif]

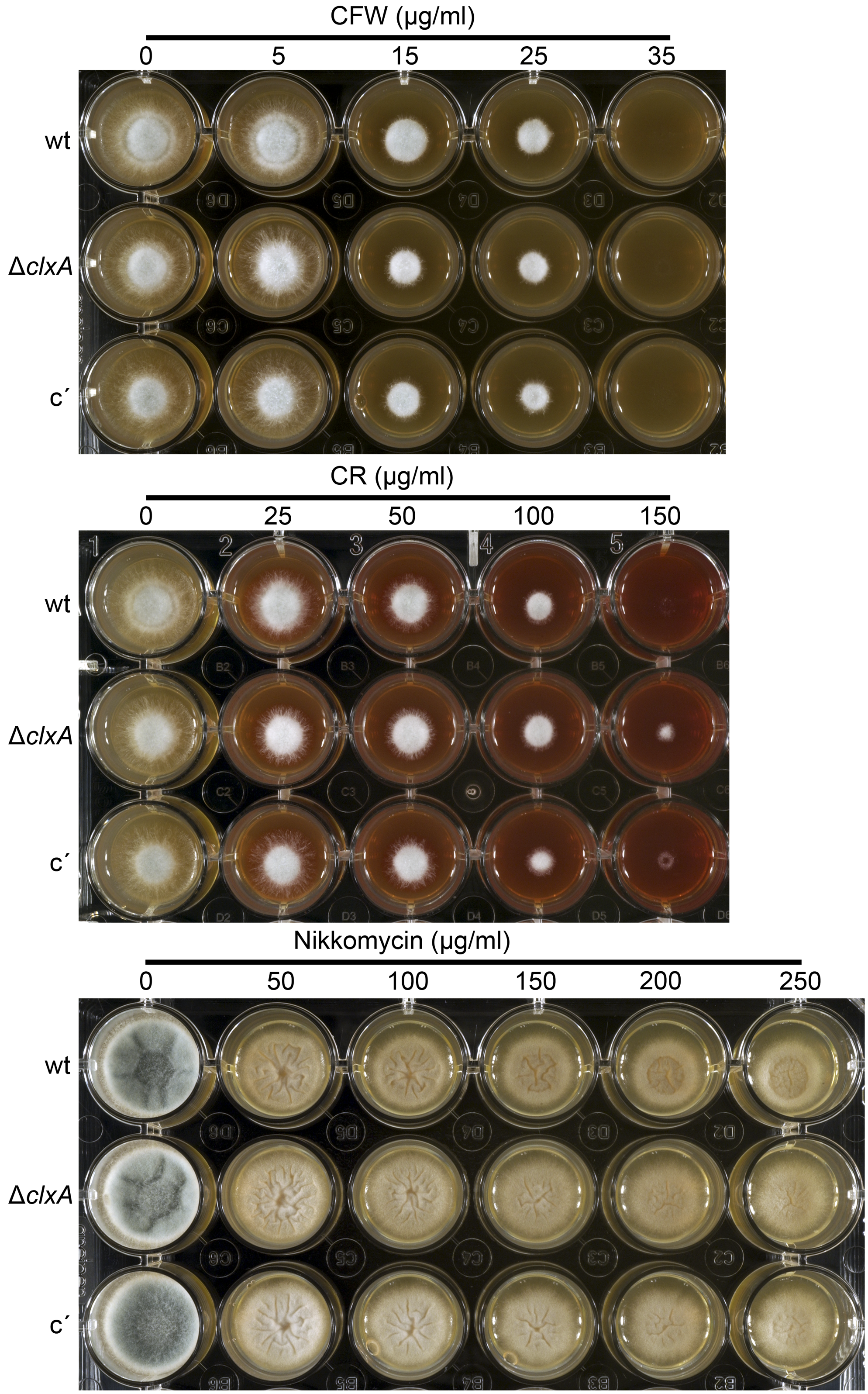

Supplement: Figure S4 — Calnexin is not required under cell wall stress conditions. Sensitivity to Congo red (CR), nikkomycin, or calcofluor white (CFW) was determined by spotting equal amounts of conidia onto the center of a plate of IMA containing each compound at the indicated concentrations and monitoring radial growth for 24 hours (CR and CFW) or 36 hours (Nikkomycin) at 37°C. The ΔclxA strain phenocopies wt at all concentrations of each cell wall stress-inducing agent. (TIF) [file pone.0028865.s004.tif]

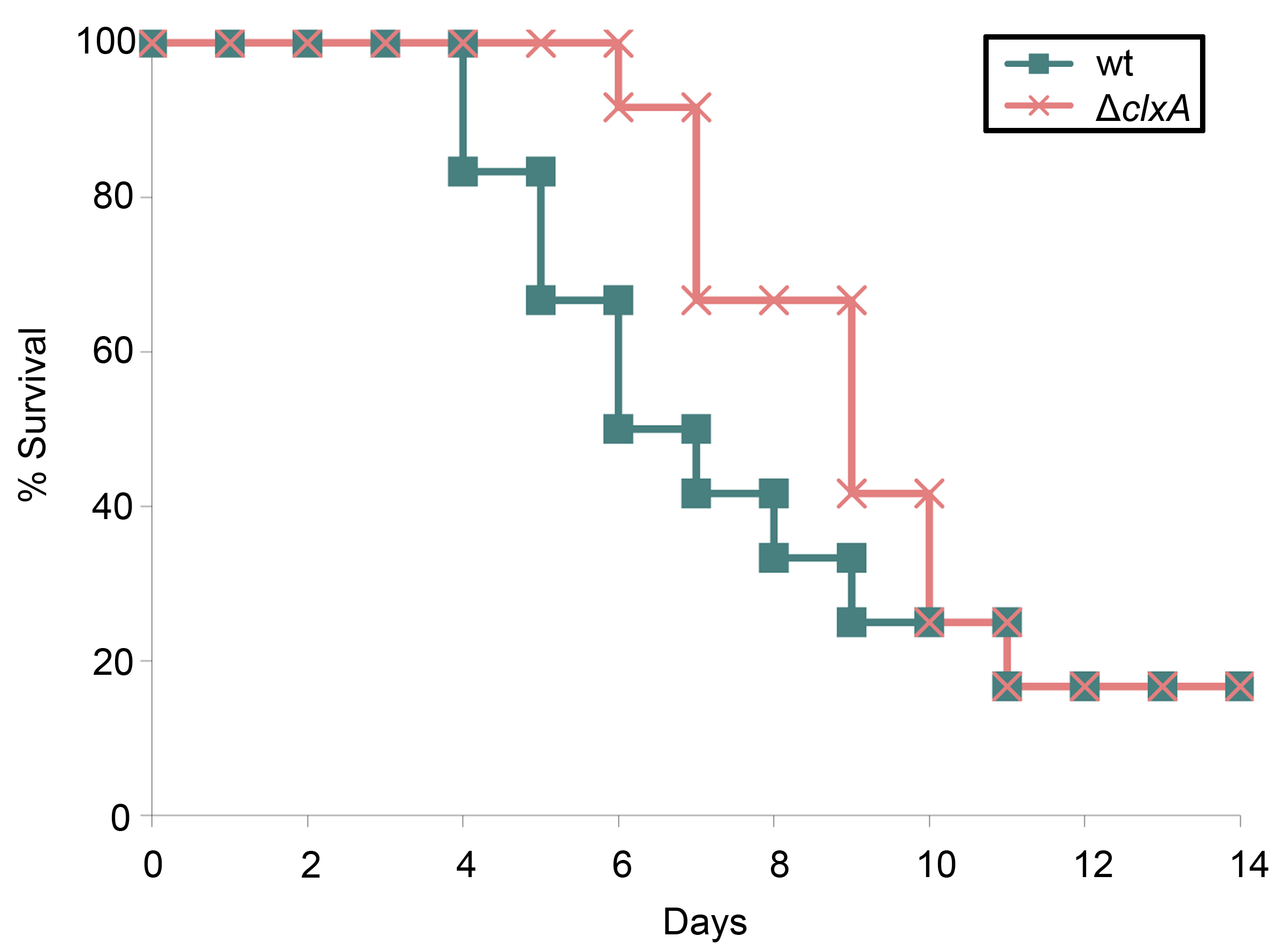

Supplement: Figure S5 — Calnexin is dispensable for A. fumigatus virulence. Groups of 12 CF-1 outbred mice were immunosuppressed with cyclophosphamide and triamcinolone acetonide and inoculated with 2×10^5^ conidia as described in Materials and Methods. Pulmonary fungal infections were confirmed in all mice that died by plating lung tissue for fungal growth. One of the four mock-infected control mice died on day +10 of a bacterial infection. The virulence of the ΔclxA mutant was statistically indistinguishable from that of wt. (TIF) [file pone.0028865.s005.tif]

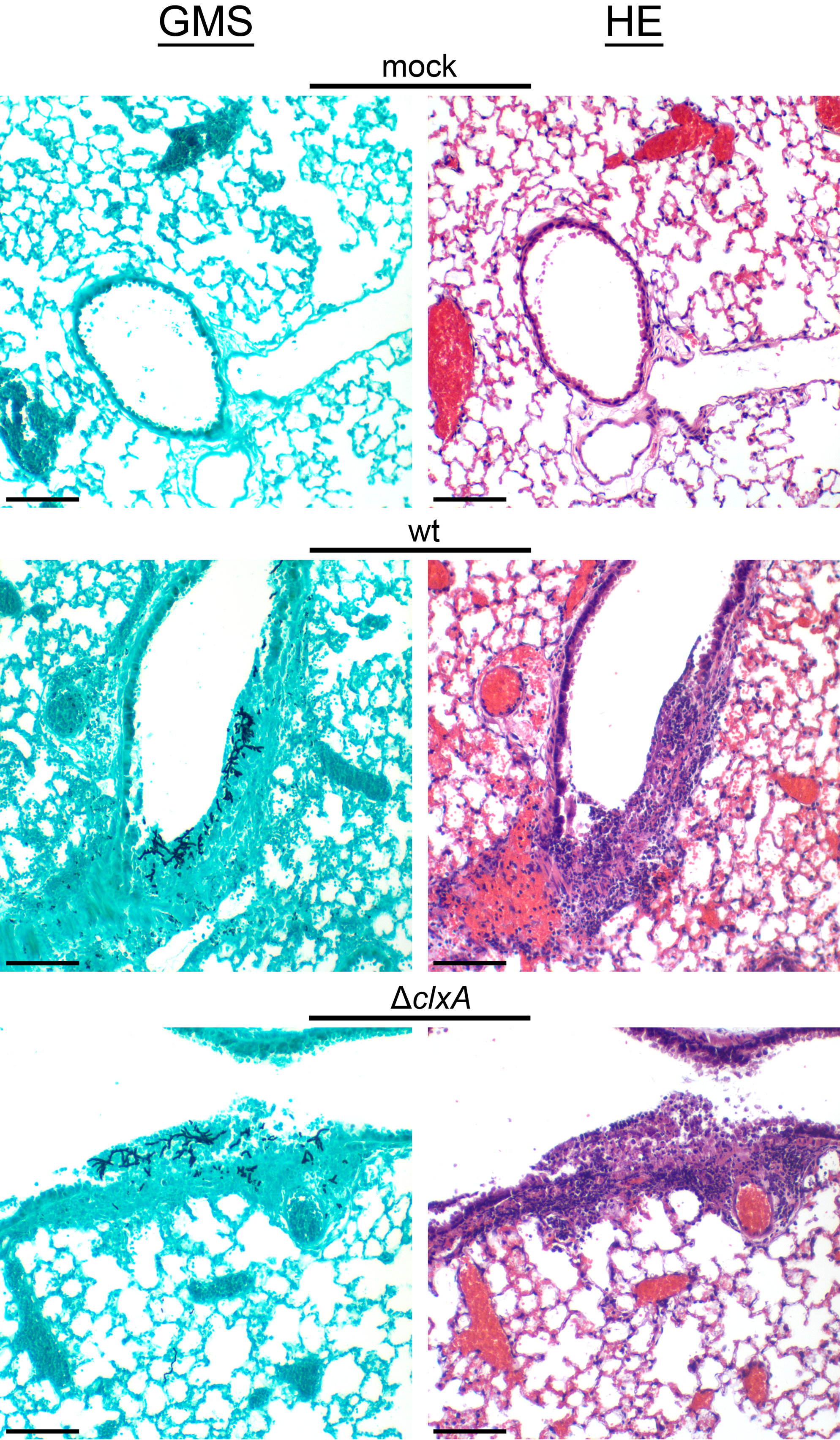

Supplement: Figure S6 — Histopathology of infected lung tissue. Using the neutropenic immunosuppression model, mice were infected with 2×10^5^ conidia in a separate experiment and sacrificed on day +3, as described in Materials and Methods. The lungs were sectioned at 5 µm and stained with hematoxylin and eosin (HE) or Grocott methenamine silver (GMS). Comparable levels of fungal growth and inflammation were observed in both wt- and ΔclxA-infected mice, resulting in similar amounts of bronchiolar erosion and migration of the hyphae across the airway wall. Microscopic examinations were performed on an Olympus BH-2 microscope and imaging system using Spot software version 4.6. Scale bar represents 100 µm. (TIF) [file pone.0028865.s006.tif]
